# Is chronic kidney disease associated with osteoarthritis? The United States national health and nutrition examination survey 2011–2020

**DOI:** 10.1186/s12882-024-03672-1

**Published:** 2024-07-25

**Authors:** Kuiliang Gao, Chao Zhang, Yifan Zhang, Longyao Zhang, Jiankang Xu, Hongfei Xue, Lingling Jiang, Jinwei Zhang

**Affiliations:** 1 Orthopedics Department, The First Teaching Hospital of Tianjin University of Traditional Chinese Medicine, Tianjin, China; 2 National Clinical Research Center for Chinese Medicine Acupuncture and Moxibustion, Tianjin, China; 3 Acupuncture Department, The First Teaching Hospital of Tianjin University of Traditional Chinese Medicine, Tianjin, China; 4 Tianjin Academy of Traditional Chinese Medicine Affiliated Hospital, Tianjin, China

**Keywords:** Chronic kidney disease, Creatinine, Osteoarthritis, NHANES

## Abstract

**Objective:**

Chronic kidney disease (CKD) and osteoarthritis (OA) represent two frequently seen disorders among the general population, and they share several similar risk factors. The present work focused on assessing the relation of CKD with OA.

**Methods:**

This cohort study included 26,280 eligible participants aged ≥ 20 years who had valid data on CKD and OA from the National Health and Nutrition Examination Survey (NHANES) 2011–2020. The association between CKD and OA was studied by logistic regression, adjusting for demographics, body mass index (BMI), socioeconomic factors, physical activity, ever smoking, alcohol using, diabetes status and hypertension status.

**Results:**

Among the participants of this study, 26.69% of OA patients had concurrent CKD, whereas this proportion was only 13.83% among non-OA patients.CKD was related to OA[OR:2.269 (95%CI:2.266–2.271), *p* < 0.01] and the relation was of significance [OR:1.031 (95%CI:1.030–1.033),*p* < 0.01] following adjustments. In subgroup analyses based on age, the relation between osteoarthritis and chronic kidney disease remained significant, and in the subgroup analyses based on gender the previously mentioned relation between OA and CKD showed opposite directions in men [OR:0.869(95%CI0.867-0.871), *p* < 0.01] and women [OR:1.178(95%CI1.177-1.180), *p* < 0.01].

**Conclusions:**

In the present 10-year large-scale national-wide survey, OA is closely related to CKD, and women with OA showed a higher risk of developing CKD compared to men. This study suggests that the relationship between OA and CKD deserves further investigation, and we suggest that patients with OA need to pay extra attention to their own kidney health.

**Supplementary Information:**

The online version contains supplementary material available at 10.1186/s12882-024-03672-1.

Chronic kidney disease (CKD) and osteoarthritis (OA) are two prevalent chronic diseases that place a heavy burden on the global healthcare system. Understanding the interrelationships between these diseases is essential for effective management and prevention strategies [1]. As a progressive renal impairment condition, CKD may lead to malnutrition, end-stage kidney disease, accelerated cardiovascular disease progression [2], disturbance of bone mineral metabolism, acute kidney injury, and an elevated risk of mortality [3]. Currently, the global prevalence of CKD is estimated to be approximately 11–13% [4], which has imposed substantial burdens on public health and economies [5].Studies [6] from various regions worldwide have demonstrated that the incidence and prevalence of CKD show an increasing trend.

Osteoarthritis (OA) represents a common musculoskeletal disease with the feature of articular cartilage degeneration, synovial membrane alterations, and subchondral bone changes [7]. Its primary symptom is joint pain, and the most commonly affected joints are the knee, hip, and ankle. Symptomatic osteoarthritis is predicted to affect 27–31 million of the US population alone [8]. Globally, it is estimated that there are 250 million individuals suffering from knee OA [9]. Due to population aging, the prevalence of hypertension, diabetes, and obesity, and the incidence rate of OA are steadily increasing [10].In addition to the direct influence of factors such as articular cartilage degeneration and force line deviation, OA is also associated to a large extent with endoenvironmental factors such as metabolic disorders, inflammation, and cellular molecular levels. Both CKD and OA are among the top 30 contributors to Years Lived with a Disability (YLD) [11].Research has suggested an increasing comorbidity between the two conditions, with the majority of patients being asymptomatic and falling into the low-to-moderate-risk category. [12]OA patients share some common risk factors with CKD, such as advanced age [13, 14], long-time application of nonsteroidal anti-inflammatory drug (NSAID) [15, 16], high blood pressure [17, 18], and overweight/obesity [19, 20]. Additionally, OA is prevalent among CKD patients undergoing dialysis [21]. Consequently, it is of paramount importance to gain an in-depth understanding of the reciprocal interactions between CKD and OA, which will pave the way for more comprehensive management of both diseases [22, 23]. The present work focused on investigating and exploring the relation of OA with CKD utilizing the 2011–2020 National Health and Nutrition Examination Survey (NHANES) data in the USA.

## Method

The NHANES is the research initiated by the National Center for Health Statistics (NCHS) in the United States, focusing on assessing health and nutritional status among the American. To ensure the representativeness of participants in this study, a multi-stage, stratified and cluster probability sampling design was applied in study organization. The Ethics Review Board of NCHS approved the NHANES research. Furthermore, the adult participants provided informed consents before participating in the survey.

**Fig. 1:**
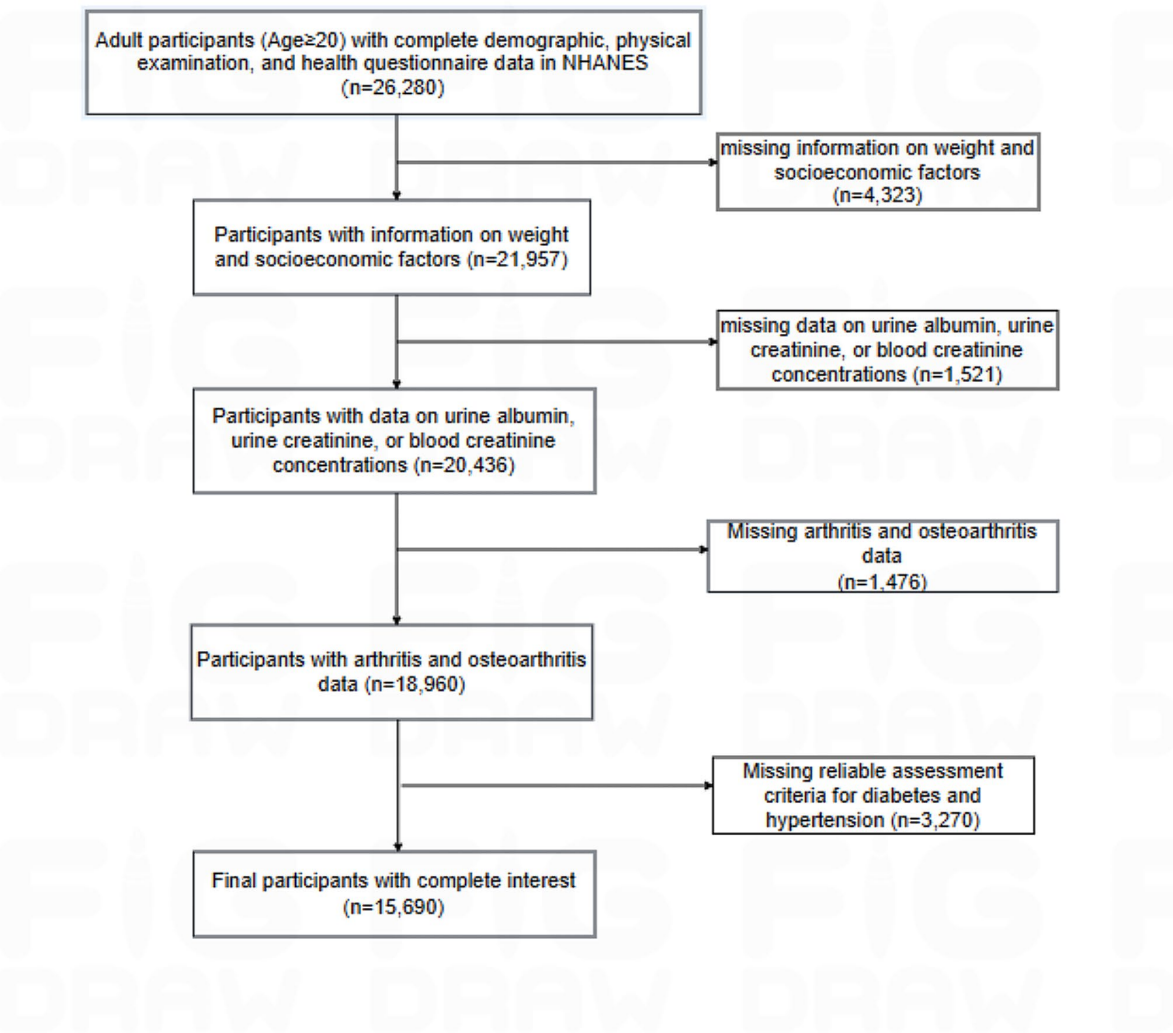
Flowchart of dataset combination

Demographic data, laboratory results, examination records, together with questionnaire responses from NHANES 2011–2020 were utilized. Adult participants (Age ≥ 20) with complete demographic, physical examination, and health questionnaire data were recruited (*n* = 26,280). Participants below were excluded: [1] participants with unavailable information on weight and socioeconomic factors (education level, family income level) (*n* = 4,323); [2] participants with missing data on urine albumin, urine creatinine, or blood creatinine concentrations, which prevented the assessment of the kidney status (*n* = 1,521); [3] participants with incomplete, unreliable, or uncertain arthritis data (*n* = 1,476); and [4] participants without reliable assessment criteria for diabetes and hypertension (*n* = 3,270). Ultimately, 15,690 participants were included in this study.(Fig. 1).

### Diagnosis of OA

The OA status was obtained through the answering the following two questions: “Did a doctor or healthcare professional ever tell you that you have arthritis?” and “What type of arthritis was it?”

According to the answers to the above questions, participants were classified as with and without OA groups.

### CKD definition

In CKD assessment, the CKD Epidemiology Collaboration (CKD-EPI) equation [24] was utilized for calculating estimated glomerular filtration rate (eGFR).CKD stage was graded in line with KDIGO guidelines [25]. Patients with CKD were categorized into two groups based on the calculated results and disease stage, where Stages 1–3 were considered early-stage CKD, whereas Stages 4–5 were categorized as middle-to-late-stage CKD.

### Covariates

Covariates encompassed demographic data, laboratory results, and survey responses. Demographic data included age, sex, ethnicity, education and family income. Ethnicity was categorized into four groups based on the self-reported survey data, including non-Hispanic White, non-Hispanic Black, Mexican American, and other. Education was divided as categories of high school or below, college, and college graduate or above. Family income was classified as three groups, namely, 130% or lower, 131-338%, and 339% or higher based on the family income-to-poverty ratio. Physical activity was classified into inactive and vigorous activity groups according to whether the weekly MEC value exceeded 600. In the questionnaire survey, individuals who smoked more than 100 cigarettes in the past were classified as smokers, and those who consumed an average of 4 or more drinks per day in the past year were classified drinkers.

The age of participants was divided as young (20–39 years), middle-aged (40–59 years), and elderly (≥ 60 years).

Additionally, stringent criteria were applied in defining diabetes and hypertension. To be specific, diabetes was deemed to be the self-reported diagnosis by a doctor, fasting blood glucose level exceeding 7.0 mmol/L, or an HbA1c level exceeding 6.5%. The blood pressure was measured thrice on average, and blood pressure was considered high with systolic pressure exceeding 140 mmHg or diastolic pressure exceeding 90 mmHg. We considered participants without blood pressure measurement data who reported taking prescription of antihypertensive medication to “Are you currently taking prescription medication” in clinical history to have high blood pressure.

### Statistical analysis

IBM SPSS Statistics 26 was employed for statistical analysis. Descriptive statistics were employed to present data, to be specific, normally distributed or skewed data were presented as mean ± standard deviation, and categorical variables were represented by counts and percentages. Complex sample weights were utilized to estimate population characteristics, BMI, and total incidence of diabetes, hypertension and arthritis.

Subsequently, covariates with statistical significance were added in a multivariate linear regression model for analysis.In this study, OA served as the predictor variable in the analysis, while CKD was the outcome variable in the associative research.The relation of CKD with OA was further explored after adjusting for covariates, and effect sizes (β) along with their 95% confidence intervals (CIs) were calculated. To enhance result accuracy, we conducted age and sex-stratified subgroup analyses.

To evaluate the performance of indicators, *p* < 0.05 (two-tailed) stood for statistical significance.

## Results

The present work included altogether 15,690 qualified subjects, their mean age was 48.48 (± SE 16.95), and 49.00% of them were males. These samples represented 180,743,385 non-institutionalized adults in the United States, including 15.83% of adults suffering from CKD and 15.50% experiencing OA. Table 1 displays the demographic and baseline characteristics of the OA group versus non-OA group. Clearly, OA group had markedly older age (*p* < 0.01) than those without OA. The proportion of female patients with OA (66.10%) was higher than males (33.90%). Non-Hispanic white individuals showed a higher proportion among arthritis patients than those without (81.83% vs. 63.92%). Besides, the smoking, physical inactivity, hypertension and diabetes rates increased among OA patients (all *p* < 0.01). Which the most important is the proportion of CKD among OA patients was higher than that among non-OA individuals (26.69% vs. 13.83%).

**Table 1:** Demographic and baseline characteristics of OA group versus non-OA group

Items	All participants	In OA group	In Non-OA group	*P* value
Age*(Years, mean ± SD)	48.48 ± 16.95	60.89 ± 16.69	46.06 ± 15.95	< 0.01
BMI*	29.54 ± 6.99	30.532 ± 7.492	29.356 ± 6.879	< 0.01
Gender,%				< 0.01
Men	49.00	33.90	51.77	
Women	51.00	66.10	48.23	
Race,%				< 0.01
Mexican Americans	7.93	3.12	8.81	
Non-Hispanic White	66.70	81.83	63.92	
Non-Hispanic Black	10.63	6.40	11.41	
Others	14.74	8.66	15.86	
Education level,%				< 0.01
High school or below	12.54	10.32	12.95	
Some College	23.30	23.42	23.28	
College graduate or above	64.16	66.27	63.77	
Poverty to income ratio,%				< 0.01
≤ 130%	20.91	17.09	21.61	
131-338%	33.56	34.40	33.41	
≥ 339%	45.53	48.51	44.99	
Ever smoking,%				< 0.01
Yes	43.77	51.09	42.43	
No	56.18	48.81	57.53	
Don’t know&Refused	0.04	0.10	0.03	
Alcohol using,%				< 0.01
Yes	14.51	6.74	15.94	
No	57.59	60.94	56.98	
Don’t know&Refused	27.90	32.32	27.08	
Diabetes status,%				< 0.01
Yes	14.55	22.68	13.06	
No	85.45	77.32	86.94	
Hypertension status,%				< 0.01
Yes	41.70	68.68	36.75	
No	58.30	31.32	63.25	
CKD status,%				< 0.01
Yes	15.83	26.69	13.83	
No	84.17	73.31	86.17	
CKD stages,%				< 0.01
1-3stages	15.23	25.57	13.33	
4-5stages	0.60	1.13	0.50	
Non-CKD	84.17	73.31	86.17	
Physical activity,%				< 0.01
Inactive	43.47	33.52	45.30	
Vigorous	34.94	35.27	34.88	
Don’t know&Refused	21.58	31.21	19.82	
OA status,%				< 0.01
Yes	15.50	-	-	
No	84.50	-	-	

BMI: body mass index; CKD: Chronic kidney disease; OA: Osteoarthritis

*Figures are expressed as mean ± standard error (for mean age, BMI), other figures are expressed as percent

Table 2 displays the logistic regression looking at the relationship between OA as the predictor and CKD stages as the outcome.On the whole, CKD was significantly related to OA [OR 2.269 (95% CI: 2.266–2.271), *p* < 0.01], and moderate-to-severe CKD was more significantly related to OA [OR 2.622 (95% CI: 2.610–2.634), *p* < 0.01]. This correlation was still significant after adjusting for demographic factors, BMI, socioeconomic factors, smoking status, alcohol consumption, physical activity, diabetes, and hypertension [OR 1.031 (95% CI: 1.030–1.033), *p* < 0.01]. Moreover, the relation between moderate-to-severe CKD and OA became even more pronounced [OR 1.178 (95% CI: 1.173–1.184), *p* < 0.01]. As revealed by Supplementary Table 1, after final model adjustment, a significant correlation was observed in both male and female CKD patients, with males showing a decreased risk compared with females [OR 0.799 (95% CI: 0.798–0.799)]. Those aged 60 years and older exhibited an apparently increased CKD risk in comparison with the 20–39 years group [OR 3.706 (95% CI: 3.700-3.712)].Furthermore, considering the impact of osteoporosis data on the results, and due to the absence of osteoporosis data for certain years in the NHANES database, we performed a regression analysis by adding osteoporosis status as a new covariate based on the years with available osteoporosis data in model 4. The results indicated a strong correlation between CKD and OA, whether in stages 1–3 CKD [OR 1.065, (95% CI:1.063–1.066) *P* < 0.01] or in stages 4–5 CKD [OR 1.446, (95% CI:1.436–1.45 *P* < 0.01]. The findings of this logistic regression analysis are presented in Supplementary Table 2.

**Table 2:** Association between chronic kidney disease status and osteoarthritis

	Crude OR (95%CI)	Model1,OR (95%CI)	Model2,OR(95%CI)	Model3,OR (95%CI)	Model4 ,OR(95%CI)
Total CKD stages					
OA	2.269(2.266–2.271)	1.139(1.138–1.140)	1.154(1.152–1.155)	1.097(1.096–1.098)	1.031(1.030–1.033)
Non-OA	Ref	Ref	Ref	Ref	Ref
1−3CKD stages					
OA	2.255(2.253–2.258)	1.136(1.134–1.137)	1.149(1.148–1.150	1.091(1.090–1.093)	1.028(1.027–1.029)
Non-OA	Ref	Ref	Ref	Ref	Ref
4−5CKD stages					
OA	2.622(2.610–2.634)	1.258(1.252–1.265)	1.316(1.310–1.323)	1.308(1.301–1.314)	1.178(1.173–1.184)
Non-OA	Ref	Ref	Ref	Ref	Ref

Logistic regression models:

Model 1: Adjusted for age, gender and race

Model 2: Further adjusted for socioeconomic factors including education level and poverty to income ratio

Model 3: Further adjusted for physical activity, alcohol using, ever cigarette smoking and obesity

Model 4: Further adjusted for diabetes and hypertension

Table 3 presents the results of stratified analyses, showing an increasing correlations of OA and CKD with age. The relation of OA with CKD in final model was as follows: for the 40–59 years group [OR = 1.152 (95% CI: 1.149–1.155), *p* < 0.01] and for females with OA [OR = 1.178 (95% CI: 1.177–1.180), *p* < 0.01].

**Table 3:** Subgroup analysis of the association of chronic kidney disease and osteoarthritis

	OR(95%CI)	*P* value
Gender		
Men		
OA	0.869(0.867–0.871)	< 0.01
Non-OA	Ref	
Women		
OA	1.178(1.177–1.180)	< 0.01
Non-OA	Ref	
Age(year)		
20–40		
OA	0.756(0.756–0.761)	< 0.01
Non-OA	Ref	
40–60		
OA	1.152(1.149–1.155)	< 0.01
Non-OA	Ref	
≥ 60		
OA	1.013(1.011–1.014)	< 0.01
Non-OA	Ref	

All data were adjusted for gender (except gender-specific estimates), age (except age-specific estimates), race, education level and poverty to income ratio, physical activity, alcohol using, ever cigarette smoking, obesity, diabetes and hypertension

## Discussion

In this study, the nationally representative data from NHANES during 2011–2020 were utilized to elucidate the close association between OA and CKD. Furthermore, even after adjusting for common factors for OA and CKD (like age, sex, ethnicity, physical activity, socioeconomic factors, obesity, previous smoking status, alcohol consumption, hypertension, and diabetes), the association remained statistically significant. Given the sufficient sample size and rigorous quality control measures employed, our analysis was considered reliable. Our findings in this study indicated a significant association between OA and CKD, even after adjusting for risk factors such as diabetes, hypertension, age, and obesity. Typically, the association was particularly strong with advanced-stage CKD (stages 4–5). Specifically, in our study, approximately 26.69% of OA patients developed concurrent CKD, whereas this proportion was only 13.83% in non-OA patients. These findings prompt us to conduct further discussion on the potential underlying factors for this interrelationship. In summary, we posit a certain correlation between OA and CKD, while metabolic dysfunction and inflammation [26], potential associations in the molecular cell field and effects of hormone levels, physical inactivity [27], hypertension, and diabetes [28] may be the potential contributors to this interrelationship.

First, inflammation may play a critical role in the interaction between OA [29]and CKD [30]. As a common inflammatory disease, the inflammatory mediators of OA are thought to be released by the cartilage, bone, and synovium [31]. However, the inflammatory symptoms of OA are not restricted to the diseased joints, and studies have suggested that several inflammatory mediators (such as IL-1β and IL-6) are up-regulated in OA than in healthy serum [32]. In OA, inflammatory mediators can be released into the bloodstream, which may induce or accelerate exposure to systemic low-grade inflammation.The persistent low-grade inflammatory state is an important factor for the occurrence and development of CKD [26], and intrarenal changes in the microvascular system induced by chronic inflammation can further lead to renal injury [33].

Second, in the molecular cellular field, CKD is associated with OA to a certain extent. Both bone morphogenetic protein-2 (BMP-2) and transforming growth factor-beta1 (TGF-β1) play critical roles in the progression of OA [22, 34]. In OA patients, the levels of TGF-β1 and BMP-2 elevate both in the diseased joints and in the plasma [35, 36]. The overexpression of BMP-2 may lead to renal injury and hypertension in CKD patients [37], whereas the elevated concentration of TGF-β1 may be the main pathogenic factor driving glomerular and tubulointerstitial fibrosis in the kidney [38, 39]. Based on the above results, we can speculate that overexpression of TGF-β1 and BMP-2 in OA patients may lead to the occurrence and aggravation of CKD. Additionally, in a previous mouse model study, mice undergoing isolated medial meniscus instability surgery exhibit an increased urinary protein level and an elevated uCr/pCr ratio [40], suggesting the unexpected effects of OA induction on renal function.

Third, as suggested by epidemiological studies, women are associated with a higher risk of OA than men, and female OA patients tend to show more severe symptoms, higher pain intensity, and lower pain tolerance than male OA patients [41, 42]. This may lead to further reduction in the physical activities of female OA patients, resulting in further serious physiological damage. In an epidemiological study [43], the incidence of knee OA significantly increases in postmenopausal women, while another study notes that the lower estrogen levels are associated with chronic musculoskeletal pain in older women in the community [44]. The gender differences in CKD revealed by epidemiological data also indicate the protective effect of estrogen on the kidney, especially in premenopausal and postmenopausal women, in whom the prevalence of CKD is reported to be 4.7% and 20.1% [45]. Noteworthily, women who experience early natural menopause before the age of 45 are at an increased risk of developing CKD [46]. According to these studies, it is reasonable to speculate that the higher estrogen levels play a certain positive role in preventing the development of OA and CKD, but female OA patients probably have low estrogen levels or are in the menopausal status, and this hormonal status may lead to an increased risk of CKD. Furthermore, genetic studies indicate that OA patients may have specific up-regulation of sex hormone-binding globulin (SHBG) [47]. In another study, the higher SHBG level is associated with the reduced risk of CKD and the improved renal function in men, but not in women, indicating that high SHBG levels in male OA patients may have some protective effect on CKD [48]. The above findings may explain the results of our sex-stratified analysis suggesting that women with OA had a higher risk of CKD while men with OA had a lower risk of CKD.

At last, in the age-stratified subgroup analysis in this study, the elderly OA patients exhibited an increased risk of CKD. Lack of physical exercises, diabetes, and hypertension may be responsible for this outcome. OA patients probably reduce their physical activities due to joint injuries, pain, or concerns about worsening their condition, and such lack of physical activities may facilitate physiological damage, like endothelial dysfunction [49]. Meanwhile, this result also aligns with previous research indicating that the elderly are associated with an increased risk of CKD, which is often related to hypertension and diabetes [50, 51].

Nonetheless, several limitations should be noted in this research. Firstly, the NHANES used in the present work was a cross-sectional survey and lacked longitudinal follow-up data. Given its retrospective nature, more studies are needed for establishing the casual relation of OA with CKD. Secondly, CKD prevalence in this study was determined using a single measurement of Scr and GFR levels, which might potentially lead to either an underestimation or overestimation of CKD prevalence. Additionally, the NHANES relied on self-reported questionnaires to collect certain medication-related variables, which might introduce self-report and recall biases. Thirdly, this study did not consider medication usage. For example, data regarding NSAIDs usage from 2011 to 2020 could not be obtained from NHANES. Therefore, future studies should continue to investigate how the different drug effects would affect the association between CKD and OA.

## Conclusion

The results of this study, based on a nationally representative survey, reveal a strong association between CKD and OA. The likelihood of CKD is significantly higher in patients with OA compared to those without, particularly among women. We suggest that OA be considered a predictor of CKD. Alongside other predisposing factors, OA should be taken into account in annual CKD screening protocols.

### Electronic supplementary material

Below is the link to the electronic supplementary material.

Supplementary Material 1

Supplementary Material 2

## Data Availability

The data that support the findings of this study are available in NHANES 2011-2020. These data were derived from the following sources available in public domain https://www.cdc.gov/nchs/nhanes/index.htm.
