# Supplementary material for: Is chronic kidney disease associated with osteoarthritis? The United States national health and nutrition examination survey 2011–2020
Source: BMC Nephrol. 2024 Jul 25;25:236. doi: 10.1186/s12882-024-03672-1 (PMC11274754; doi:10.1186/s12882-024-03672-1)
Supplement: Supplementary file 1 — Supplementary Material 1 [file 12882_2024_3672_MOESM1_ESM.docx]

Supplementary Table 1：The odds ratio of all influencing factors on chronic kidney disease status in Model4.

| Characteristics | OR(95%CI) | P value |
| --- | --- | --- |
| OA |  |  |
| OA patients | 1.031（1.03-1.033） | <0.01 |
| Non-OA patients | - |  |
| Age |  |  |
| ≥60 | 3.706（3.7-3.712） | <0.01 |
| 40-59 | 1.238（1.236-1.24） |  |
| 20-39 | - |  |
| Gender |  |  |
| Men | 0.799（0.798-0.799） | <0.01 |
| Women | - |  |
| Race |  |  |
| Mexican Americans | 1.272（1.269-1.275） | <0.01 |
| Non-Hispanic White | 1.26（1.258-1.262） |  |
| Non-Hispanic Black | 1.19（1.188-1.192） |  |
| Others | - |  |
| Education level |  |  |
| High school or below | 1.002（1.001-1.004） | <0.01 |
| Some College | 0.967（0.966-0.968） |  |
| College graduate or above | - |  |
| Poverty to income ratio |  |  |
| ≥339% | 1.748（1.746-1.751） | <0.01 |
| 131%-338% | 1.464（1.462-1.465） |  |
| ≤130% | - |  |
| Physical activity |  |  |
| Don’t know&Refused | 1.37（1.368-1.372） | <0.01 |
| Vigorous | 1.128（1.127-1.129） |  |
| Inactive | - |  |
| BMI |  |  |
| ≥30 | 0.936（0.935-0.937） | <0.01 |
| 25-30 | 0.856（0.855-0.857） |  |
| ≤25 | - |  |
| Ever smoking |  |  |
| Don’t know&Refused | 1.462（1.436-1.489） | <0.01 |
| Yes | 1.037（1.036-1.038） |  |
| No | - |  |
| Alcohol using |  |  |
| Don’t know&Refused | 1.224（1.223-1.226） | <0.01 |
| Yes | 1.078（1.076-1.079） |  |
| No | - |  |
| Diabetes |  |  |
| Yes | 2.639（2.636-2.642） | <0.01 |
| No | - |  |
| Hypertension |  |  |
| Yes | 2.13（2.127-2.132） | <0.01 |
| No | - |  |
