# Supplementary material for: Is chronic kidney disease associated with osteoarthritis? The United States national health and nutrition examination survey 2011–2020
Source: BMC Nephrol. 2024 Jul 25;25:236. doi: 10.1186/s12882-024-03672-1 (PMC11274754; doi:10.1186/s12882-024-03672-1)
Supplement: Supplementary file 2 — Supplementary Material 2 [file 12882_2024_3672_MOESM2_ESM.docx]

Supplementary Table 2：

Supplementary Table 2 shows the results of logistic regression analysis with osteoporosis added as a new covariate on top of Model 4.

| Characteristics | 1-3CKD stage | | 4-5CKD stage | |
| --- | --- | --- | --- | --- |
|  | OR(95%CI) | P value | OR(95%CI) | P value |
| OA |  |  |  |  |
| OA patients | 1.065(1.063-1.066) | <0.01 | 1.446(1.436-1.456) | <0.01 |
| Non-OA patients | - |  | - |  |
| Age |  |  |  |  |
| ≥60 | 2.423(2.419-2.427) | <0.01 | 1.702(1.691-1.714) | <0.01 |
| 40-50 | - |  | - |  |
| Gender |  |  |  |  |
| Men | 0.921(0.919-0.922) | <0.01 | 1.247(1.239-1.255) | <0.01 |
| Women | - |  | - |  |
| Race |  |  |  |  |
| Mexican Americans | 1.098(1.093-1.102) | <0.01 | 1.284(1.264-1.303) | <0.01 |
| Non-Hispanic White | 1.403(1.400-1.406) |  | 1.222(1.210-1.233) |  |
| Non-Hispanic Black | 1.175(1.171-1.179) |  | 2.407(2.383-2.432) |  |
| Others | - |  | - |  |
| Education level |  |  |  |  |
| High school or below | 0.929(0.927-0.931) | <0.01 | 1.002(0.994-1.010) | >0.5 |
| Same College | 0.889(0.888-0.891) |  | 0.698(0.693-0.703) |  |
| College graduate or above | - |  | - |  |
| Poverty to income ratio |  |  |  |  |
| ≥339% | 1.694(1.691-1.698) | <0.01 | 1.945(1.929-1.961) | <0.01 |
| 131%-338% | 1.495(1.492-1.497) |  | 1.129(1.120-1.137) |  |
| ≤130% | - |  | - |  |
| Physical activity |  |  |  |  |
| Don’t know&Refused | 1.468(1.465-1.471) | <0.01 | 1.820(1.806-1.834) | <0.01 |
| Vigorous | 1.247(1.244-1.249) |  | 0.949(0.941-0.956) |  |
| Inactive | - |  | - |  |
| BMI |  |  |  |  |
| ≥30 | 1.013(1.011-1.015) | <0.01 | 0.450(0.446-0.453) | <0.01 |
| 25-30 | 0.940(0.938-0.42) |  | 0.527(0.523-0.531) |  |
| ≤25 | - |  | - |  |
| Ever smoking |  |  |  |  |
| Don’t know&Refused | 3E-10 | >0.5 | - | - |
| Yes | 1.096(1.094-1.098) |  | 0.825(0.820-0.830) | <0.01 |
| No | - |  | - |  |
| Alcohol using |  |  |  |  |
| Don’t know&Refused | 1.206(1.204-1.208) | <0.01 | 4.000(3.972-4.027) | <0.01 |
| Yes | 0.894(0.891-0.897) |  | 0.449(0.439-0.460) |  |
| No | - |  | - |  |
| Diabetes |  |  |  |  |
| Yes | 2.607(2.603-2.612) | <0.01 | 4.079(4.054-4.105) | <0.01 |
| No | - |  | - |  |
| Hypertension |  |  |  |  |
| Yes | 2.378(2.374-2.382) | <0.01 | 6.138(6.072-6.204) | <0.01 |
| No | - |  | - |  |
| Osteoporosis |  |  |  |  |
| Yes | 1.163(1.1631-1.166) | <0.01 | 1.060(1.050-1.69) | <0.01 |
| No | - |  | - |  |
